# *Scytinostromabambusinum* sp. nov. (Russulales, Basidiomycota) in China evidenced by morphological characteristics and phylogenetic analyses

**DOI:** 10.3897/BDJ.12.e115975

**Published:** 2024-05-28

**Authors:** Xiao-Hong Ji, Bin Sun, Gang He, Qi-Biao Sun

**Affiliations:** 1 College of Pharmacy and Life Sciences, Jiujiang University, Jiujiang, China College of Pharmacy and Life Sciences, Jiujiang University Jiujiang China; 2 Key Laboratory of Watershed Ecological Process and Information of Jiangxi Province, Jiujiang, China Key Laboratory of Watershed Ecological Process and Information of Jiangxi Province Jiujiang China; 3 Jiujiang Key Laboratory of Fungal Resources Conservation and Utilization, Jiujiang, China Jiujiang Key Laboratory of Fungal Resources Conservation and Utilization Jiujiang China

**Keywords:** taxonomy, phylogeny, wood-decaying fungi

## Abstract

**Background:**

Wood-rotting fungi as an important group within the Basidiomycota are known for their ecological role in the forest ecosystem in terms of decaying living and dead trees and recycling nutrients in forest ecosystems. Many new species were revealed in the last five years. In the present study, during an ongoing study on *Scytinostroma*, a new species of *Scytinostroma* was found from China. It is described and illustrated on the basis of the morphological and phylogenetic evidence.

**New information:**

*Scytinostromabambusinum* sp. nov. is described as a new species, based on morphological and molecular evidence. It is characterised by annual, resupinate and broadly ellipsoid basidiomata with white to cream hymenophore, a dimitic hyphal structure with generative hyphae bearing simple septa, the presence of cystidioles and amyloid basidiospores measuring 5.5–7 × 4–5.3 µm. Phylogeny, based on molecular data of ITS and nLSU sequences, shows that the new species forms an independent lineage and is different in morphology from the existing species of *Scytinostroma*.

## Introduction

*Scytinostroma* Donk (Russulales, Basidiomycota) was established by Donk (1956) with *S.portentosum* (Berk. & M.A. Curtis) Donk as the type species. The species in the genus have resupinate, coriaceous basidiomata, smooth to tuberculate hymenophore and a dimitic hyphal structure with simple septa or clamps on generative hyphae, skeletal hyphae densely branched and sometimes forming dendrohyphae or dichohyphae, strongly dextrinoid and cyanophilous and the presence of cystidia, basidia tubular to uniform and subglobose to ellipsoid, smooth, thin-walled, variably amyloid basidiospores and causing white rot (Donk 1956, Bernicchia and Gorjón 2010, Wang et al. 2020, Tabish and Daniel 2021, Zhang et al. 2023). The most obvious character of this genus is the tough and leathery texture of the basidiome, as well as dextrinoid and dichotomously branched skeletal hyphae (Rattan 1974, Liu et al. 2019). After several rearrangements (Donk 1956, Gilbertson 1962, Boidin 1967, Rattan 1974, Boidin and Lanquetin 1977, Lanquetin 1984, Boidin and Lanquetin 1987, Boidin and Gilles 1988, Hjortstam 1990, Stalpers 1996) and recent discoveries of the genus (Nakasone 2008, Wang et al. 2020, Zhang et al. 2023, Li et al. 2023), so far, 42 species have been described or transferred to the genus worldwide (Donk 1956, Bernicchia and Gorjón 2010, Wang et al. 2020, Tabish and Daniel 2021, Zhang et al. 2023, Li et al. 2023). The latest molecular studies involving *Scytinostroma*, based on concatenated ITS1-5.8S-ITS2-nrLSU sequence data, have been carried out (Zhang et al. 2023, Li et al. 2023).

During a survey for wood-decaying fungi from China, two samples were collected from Jiangxi Province and their morphological characters fit *Scytinostroma* well. To confirm their taxonomic affinity and the evolutionary relationships amongst representative species of *Scytinostroma*, phylogenetic analysis was carried out, based on ITS and nLSU sequences. Both morphological and molecular data support these two samples to represent a new species. In this paper, we give an illustrated description for the new species *S.bambusinum*.

## Materials and methods

### Morphological studies

The studied specimens were deposited in the Mycological Herbarium of Jiujiang University (MHJU), China. Microscopic examination follows Dai (2010) and colour terms follow Petersen (1996). Spores were measured from sections cut from the tubes. Five percent of measurements were excluded from each end of the range and are given in parentheses. Abbreviations include IKI = Melzer’s reagent, IKI– = negative in Melzer’s reagent, KOH = 5% potassium hydroxide, CB = Cotton Blue, CB+ = cyanophilous, CB– = acyanophilous, L = mean spore length (arithmetic average of all spores), W = mean spore width (arithmetic average of all spores), Q = the L/W ratio and n = number of spores measured from the given number of specimens.

### Molecular study and phylogenetic analysis

A CTAB-based rapid plant genome extraction kit (Aidlab Biotechnologies Co., Ltd, Beijing) was used to obtain genomic DNA from dried specimens. The DNA was amplified with the following primers: ITS4 and ITS5 for ITS (White et al. 1990) and LR0R and LR7 for nLSU. The PCR procedure for ITS amplification was as follows: initial denaturation at 95°C for 3 min, followed by 35 cycles at 94°C for 40 s, 54°C for 45 s and 72°C for 1 min and a final extension of 72°C for 10 min. The PCR procedure for nLSU was as follows: initial denaturation at 94°C for 1 min, followed by 35 cycles of 94°C for 30 s, 50°C for 1 min and 72°C for 1.5 min and a final extension of 72°C for 10 min. The PCR products were purified and sequenced at the Changsha Genomics Institute, China, with the same primers. The newly-generated sequences were deposited in GenBank (Table 1).

Besides the newly-generated sequences, additional sequences downloaded from GenBank were also included for phylogenetic analyses of ITS and nLSU phylogenetic tree (Table 1, Zhang et al. (2023)). *Confertobasidiumolivaceoalbum* (Bourdot & Galzin) Jülich (Jülich 1972) and *Metulodontianivea* (P. Karst.) Parmasto (Parmasto 1968) were selected as outgroups (Larsson and Larsson 2003). The sequences were aligned using ClustalX 1.83 (Chenna et al. 2003) and alignments were curated manually in BioEdit 7.0.5.3 (Hall 1999). Prior to phylogenetic analyses, ambiguous regions at the start and the end were deleted.

Maximum Likelihood (ML), Maximum Parsimony (MP) and Bayesian Inference (BI) analyses were performed for the ITS and nLSU dataset. MP analysis was performed using PAUP* 4.0b10 (Swofford 2002) with gaps in the alignments treated as missing data. Trees were inferred using heuristic search option with TBR branch swapping and 1,000 random sequence additions. Max-trees were set to 5,000, branches of zero length were collapsed and all parsimonious trees were saved. Clade robustness was assessed using bootstrap analysis with 1,000 replicates (Felsenstein 1985). Descriptive tree statistics tree length (TL), consistency index (CI), retention index (RI), rescaled consistency index (RC) and homoplasy index (HI) were calculated for each maximum parsimonious tree generated. Sequences were also analysed using ML with RAxML-HPC2 on Abe through the CIPRES Science Gateway (www.phylo.org). BI was calculated with MrBayes3.1.2 with a general time reversible (GTR) model of DNA substitution and a gamma distribution rate variation across sites (Ronquist and Huelsenbeck 2003). MrModelTest2.3 (Nylander 2004) was used to determine the best-fit evolution model for the dataset.

The BI was conducted with MrBayes 3.2.6 in two independent runs, each of which had four chains for 10 million generations and started from random trees (Ronquist and Huelsenbeck 2003). Trees were sampled every 1,000 generations. The first 25% of the sampled trees were discarded as burn-in and the remaining ones were used to reconstruct a majority rule consensus and calculate Bayesian Posterior Probabilities (BPP) of the clades.

The three phylogenetic analyses algorithms generated nearly identical topologies for the dataset; thus, only the topology from the MP analysis is presented along with statistical values from the ML, MP and BI algorithms (Bootstrap support < 50% and Bayesian posterior probabilities < 0.9 are not shown) at the nodes. Tree was visualised in TreeView 1.6.6 (Page 1996).

## Taxon treatments

### Scytinostroma bambusinum

X.H. Ji sp. nov.

818C8DAC-1C68-54B2-B100-88F73DEE7945

853562

#### Materials

- **Type status:** Holotype. **Occurrence:** recordNumber: JXH 596; recordedBy: Ji Xiao-Hong; individualCount: 1; occurrenceID: F8ECA17A-1B7D-52C8-8C7D-855EE065E847; **Taxon:** scientificName: Scytinostromabambusinum; acceptedNameUsage: Scytinostromabambusinum X.H. Ji, 2023, sp. nov.; parentNameUsage: Scytinostroma Donk 1956; kingdom: Fungi; phylum: Basidiomycota; class: Agaricomycetes; order: Russulales; family: Peniophoraceae; genus: Scytinostroma; specificEpithet: bambusinum; taxonRank: species; verbatimTaxonRank: species; scientificNameAuthorship: X.H. Ji; **Location:** continent: Asia; country: China; stateProvince: Jiangxi; municipality: Ji'an; locality: Jinggangshan, Ji'an, Jiangxi Province, China; verbatimElevation: 918 m; locationRemarks: label transliteration: "Jiangxi, Ji'an, Jinggangshan, 16/05/2023, Ji Xiaohong"; [江西吉安井冈山, 16/05/2023, 季晓红]; georeferenceProtocol: label; **Identification:** identifiedBy: Ji Xiaohong; dateIdentified: 2023; **Event:** samplingProtocol: collecting; eventDate: 16/05/2023; **Record Level:** language: en; institutionCode: the Herbarium of Jiujiang Training Collega (JJTC); collectionCode: Fungi; ownerInstitutionCode: the Herbarium of Jiujiang Training Collega (JJTC); basisOfRecord: PreservedSpecimen
- **Type status:** Isotype. **Occurrence:** recordNumber: JXH 643; recordedBy: Ji Xiao-Hong; individualCount: 1; occurrenceID: 11545F7B-1F13-5002-8465-26139F26B467; **Taxon:** scientificName: Scytinostromabambusinum; acceptedNameUsage: Scytinostromabambusinum X.H. Ji, 2023, sp. nov.; parentNameUsage: Scytinostroma Donk 1956; kingdom: Fungi; phylum: Basidiomycota; class: Agaricomycetes; order: Russulales; family: Peniophoraceae; genus: Scytinostroma; specificEpithet: bambusinum; taxonRank: species; verbatimTaxonRank: species; scientificNameAuthorship: X.H. Ji; **Location:** continent: Asia; country: China; stateProvince: Jiangxi; municipality: Ji'an; locality: Jinggangshan, Ji'an, Jiangxi Province, China; verbatimElevation: 856 m; locationRemarks: label transliteration: "Jiangxi, Ji'an, Jinggangshan, 17/05/2023, Ji Xiaohong"; [江西吉安井冈山, 2023.05.17, 季晓红]; georeferenceProtocol: label; **Identification:** identifiedBy: Ji Xiaohong; dateIdentified: 2023; **Event:** samplingProtocol: collecting; eventDate: 17/05/2023; **Record Level:** language: en; institutionCode: the Herbarium of Jiujiang Training Collega (JJTC); collectionCode: Fungi; ownerInstitutionCode: the Herbarium of Jiujiang Training Collega (JJTC); basisOfRecord: PreservedSpecimen

#### Description

**Figure 1.**
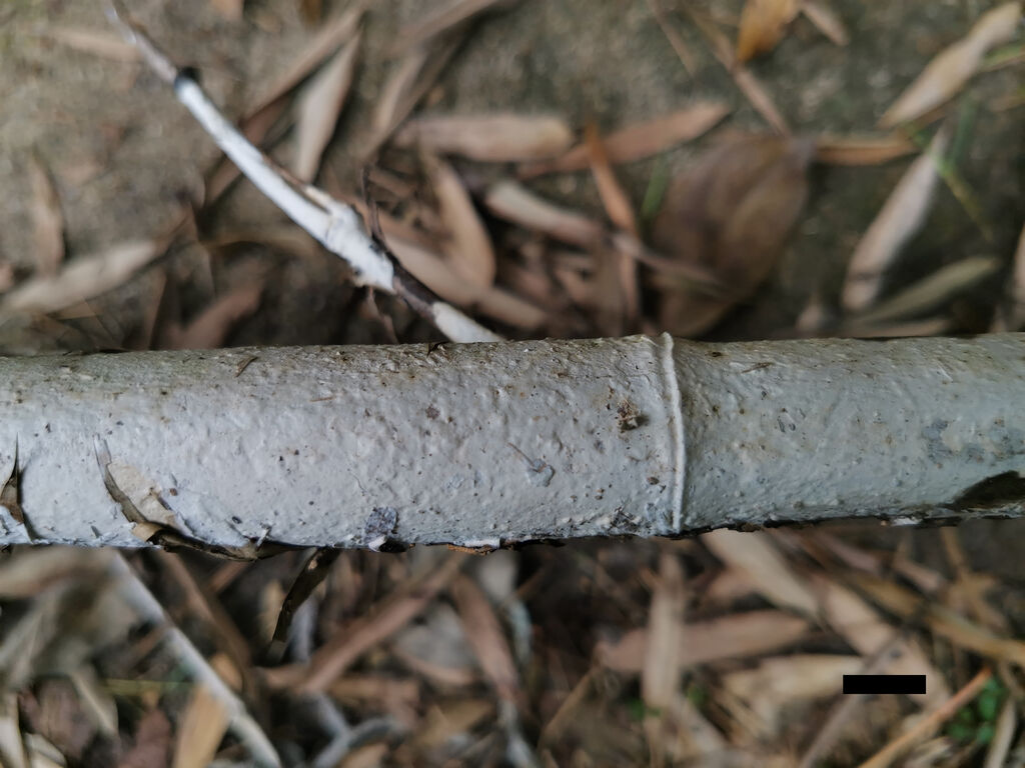
Basidiome of *Scytinostromabambusinum* (JXH 596). Scale bar: 1 cm.

Basidiomata (Fig. 1) — Annual, resupinate, coriaceous, not separable from substrate, up to 30 cm long, 4 cm wide and less than 0.3 mm thick at centre. Hymenial surface smooth to tuberculate, white to cream when fresh, cream upon drying; margin concolorous with hymenial surface, thinning out and adnate.

Hyphal structure — Hyphal system dimitic; generative hyphae simple septate, hyaline, thin-walled, rarely branched, 1.5–3 µm in diameter, IKI–, CB–; tissues unchanged in KOH; skeletal hyphae dominant, frequently branched, interwoven, thick-walled, 2–3.5 µm in diameter, cyanophilous.

Hymenium — Cystidia absent; cystidioles present, clavate, some gradually tapering to the apex, thin-walled, hyaline, smooth, 24–28 × 3–5 µm; basidia clavate, with a basal simple septum and four sterigmata, thin-walled, smooth, 20–25 × 5–8 µm; basidioles similar to basidia in shape, but slightly smaller.

**Figure 2.**
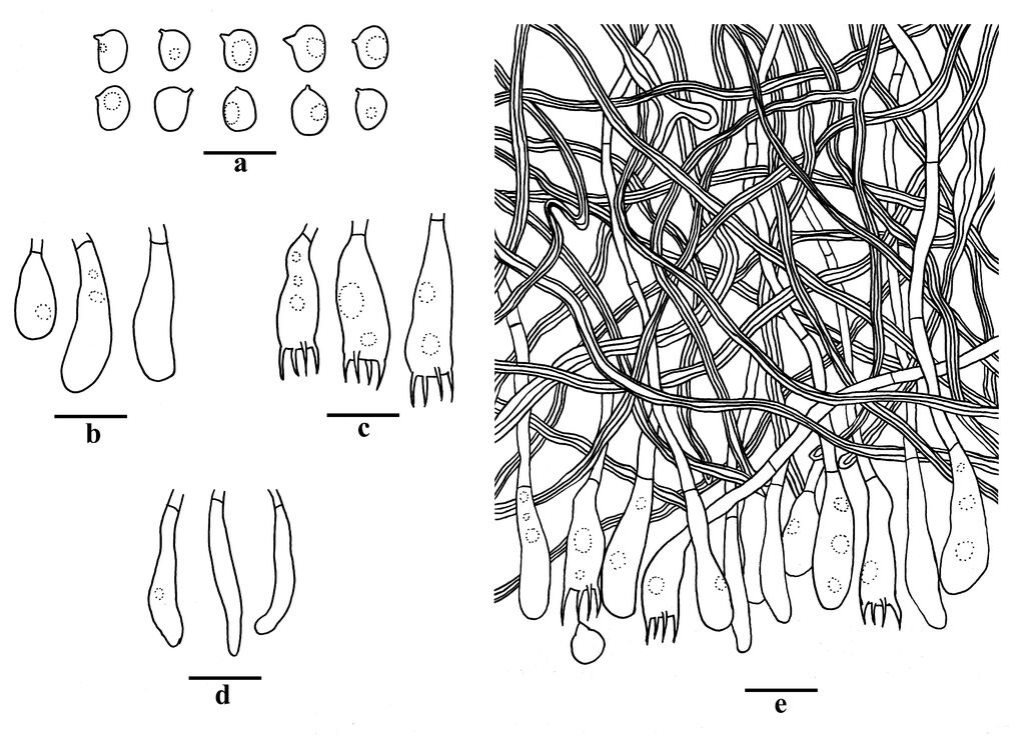
Microscopic structures of *Scytinostromabambusinum* (Holotype). **A** Basidiospores; **B** Basidioles; **C** Basidia; **D** Cystidioles; **E S**ection of basidiome. Scale bars: 10 μm.

Spores — Basidiospores broadly ellipsoid with an apiculus, hyaline, thin-walled, smooth, occasionally with one guttule, amyloid, acyanophilous, (5.3–)5.5–7(–7.3) × 4–5.3(–5.5) µm, L = 6.00 µm, W = 4.57 µm, Q = 1.28–1.31 (n = 60/1) (Fig. 2).

#### Etymology

*Bambusinum* (Lat.): refers to the species growing on dead bamboo.

## Analysis

**Figure 3.**
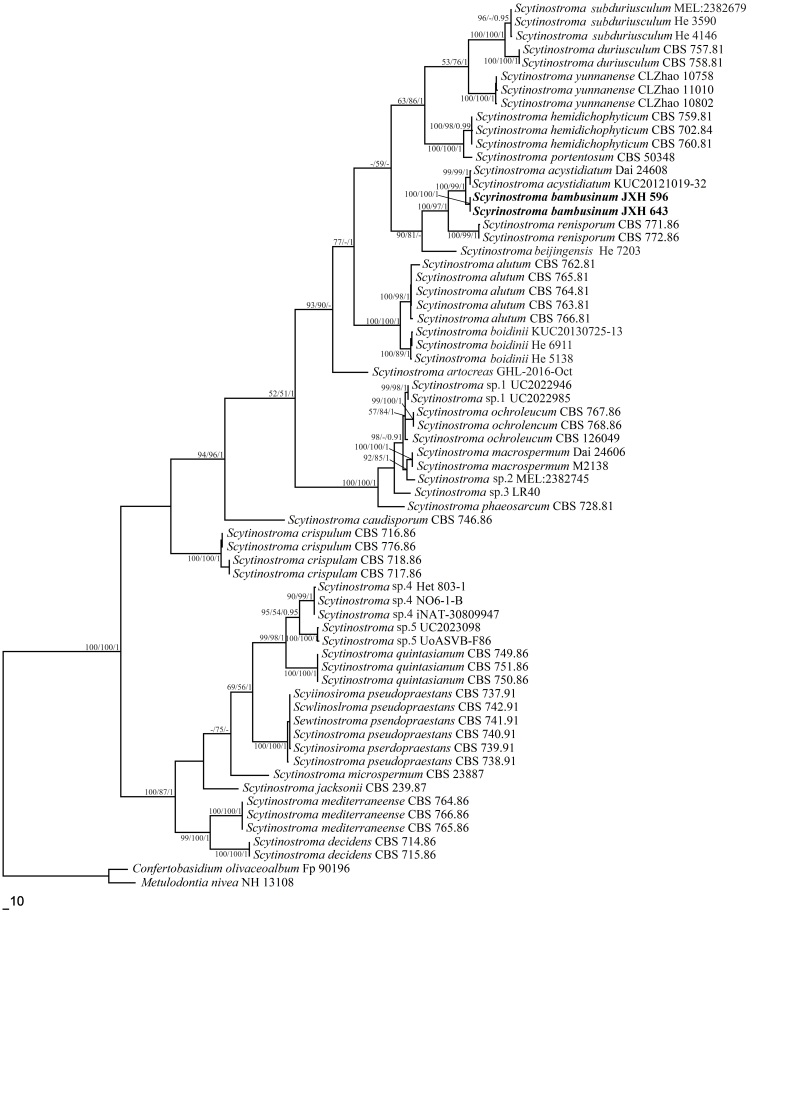
Phylogeny of *Scytinostroma* inferred from the ITS and nLSU dataset. Topology is from the MP tree and statistical values (MP/ML/BI) are indicated for each node that simultaneously received BS from MP and ML not below 50% and BPPs from BI not below 0.9.

Two newly-generated ITS and nLSU sequences of the new species are deposited at GenBank. The accession numbers of the sequences in this study are labelled in the phylogenetic tree (Fig. 3). The ITS and nLSU dataset has 66 taxa and resulted in an alignment of 1653 characters, of which 748 characters are constant, 67 are variable and parsimony-uninformative and 838 are parsimony-informative. Maximum parsimony analysis yielded 15 equally parsimonious trees (TL = 3274, CI = 0.520, HI = 0.480, RI = 0.859, RC = 0.447). Best model for the ITS and nLSU estimated and applied in the Bayesian analysis is: GTR+I+G, lset nst = 6, rates = invgamma; prset statefreqpr = dirichlet (1, 1, 1, 1). The Bayesian and ML analyses produced similar topologies compared to the MP analysis, with an average standard deviation of split frequencies = 0.009737 (BI). The phylogenetic tree (Fig. 3) shows that the two newly-sequenced specimens form a distinct lineage with full statistical supports (100% BS, 100% BP, 1.00 BPP) and this lineage occupies a separate position from known species of *Scytinostroma* (Fig. 3).

## Discussion

The new species, *Scytinostromabambusinum*, is described, based on morphological differences and molecular phylogenetic analyses in this study. The unique morphological characters and phylogeny, based on ITS and nLSU sequences (Fig. 3), show the position of the new species in the genus *Scytinostroma*.

Phylogenetically, *Scytinostromabambusinum* was grouped with *S.acystidiatum* Q.Y. Zhang, L.S. Bian & Q. Chen and *S.renisporum* Boidin, Lanq. & Gilles with a strong support (Fig. 3). However, morphologically *S.acystidiatum* differs from *S.bambusinum* by its smaller cystidioles (12–18 × 2–4 µm) and smaller basidiospores (4.7–7 × 3.5–4.7 µm; Zhang et al. (2023)). *Scytinostromarenisporum* is morphologically distinguished from *S.bambusinum* by its membranaceous to paper-like basidiomata and gloeocystidia measuring 20–35 × 6–10 µm (Boidin and Lanquetin 1987). Morphologically, *Scytinostromaalutum* Lanq., *S.arachnoideum* (Peck) Gilb. and *S.yunnanense* C.L. Zhao are similar to *S.bambusinum* by sharing amyloid basidiospores and the absence of cystidia. However, *S.alutum* differs from *S.bambusinum* by its resupinate to effuse-reflexed basidiomata with cracked hymenophore and larger basidia (40–65 × 5–6 µm, Lanquetin (1984)). *Scytinostromaarachnoideum* is separated from *S.bambusinum* by its cottony basidiomata with white rhizomorphs and smaller basidiospores (3.5–4.5 × 3–3.5 µm, Gilbertson (1962)). *Scytinostromayunnanense* differs from *S.bambusinum* by its smaller (4.5–5.5 × 4.2–5.2 µm) and subglobose to globose basidiospores (Wang et al. 2020). In addition, our new taxon, *Scytinostromabambusinum*, was collected from bamboo.

Species diversity of *Scytinostroma* in China remains poorly known, especially in south-eastern China, a hotspot of biodiversity. The new species in the present study, *Scytinostromabambusinum*, is from south-eastern China. It is possible that new taxa will be found after further investigations.

## Supplementary Material

XML Treatment for Scytinostroma bambusinum

## Figures and Tables

**Table 1.** Information on the sequences used in this study. The new species are shown in bold.

**Species**	**Specimen no.**	**Locality**	**ITS**	**nLSU**	**Literature**
*Confertobasidiumolivaceoalbum*	FP 90196	USA	AF511648	AF511648	Larsson and Larsson (2003)
*Metulodontianivea*	NH 13108	Russia	AF506423	AF506423	Larsson and Larsson (2003)
*Scytinostromaacystidiatum*	Dai 24608	China	OQ689127	OQ629351	Zhang et al. (2023)
*S.acystidiatum*	KUC20121019-32	Korea	KJ668461	−	Jang et al. (2016)
*S.alutum*	CBS 762.81	France	MH861482	MH873221	Vu et al. (2019)
*S.alutum*	CBS 763.81	France	MH861483	MH873222	Vu et al. (2019)
*S.alutum*	CBS 764.81	France	MH861484	MH873223	Vu et al. (2019)
*S.alutum*	CBS 765.81	France	MH861485	MH873224	Vu et al. (2019)
*S.alutum*	CBS 766.81	France	MH861486	MH873225	Vu et al. (2019)
*S.artocreas*	GHL-2016-Oct	USA	MH142900	MH204691	Liu et al. (2019)
*S.beijingensis*	He 7768	China	OQ731943	OQ729731	Li et al. (2023)
*S . boidinii*	KUC20130725-13	Korea	KJ668460	−	Jang et al. (2016)
*S.boidinii*	He 5138	China	MK625572	MK625497	Li et al. (2023)
*S.boidinii*	He 6911	China	OQ731934	OQ729724	Li et al. (2023)
***S.bambusinum***	**JXH 643**	**China**	**OR510627**	**PP660872**	**Present study**
***S.bambusinum***	**JXH 596**	**China**	**OR510628**	**PP660873**	**Present study**
*S.caudisporum*	CBS 746.86	Gabon	MH862030	NG073580	Vu et al. (2019)
*S.crispulum*	CBS 716.86	Reunion	MH862013	MH873703	Vu et al. (2019)
*S.crispulum*	CBS 717.86	France	MH862014	MH873704	Vu et al. (2019)
*S.crispulum*	CBS 718.86	France	MH862015	MH873705	Vu et al. (2019)
*S.crispulum*	CBS 776.86	France	MH862053	MH873741	Vu et al. (2019)
*S.decidens*	CBS 714.86	France	MH862011	MH873701	Vu et al. (2019)
*S.decidens*	CBS 715.86	France	MH862012	MH873702	Vu et al. (2019)
*S.duriusculum*	CBS 757.81	France	MH861477	MH873216	Vu et al. (2019)
*S.duriusculum*	CBS 758.81	France	MH861478	MH873217	Vu et al. (2019)
*S.hemidichophyticum*	CBS 702.84	Belgium	MH861818	MH873509	Vu et al. (2019)
*S.hemidichophyticum*	CBS 759.81	France	MH861479	MH873218	Vu et al. (2019)
*S.hemidichophyticum*	CBS 760.81	France	MH861480	MH873219	Vu et al. (2019)
*S.jacksonii*	CBS 239.87	Canada	MH862071	MH873759	Vu et al. (2019)
*S.macrospermum*	Dai 24606	China	OQ689126	OQ629350	Zhang et al. (2023)
*S.macrospermum*	M2138	Japan	LC327052	−	Ogura‐Tsujita et al. (2018)
*S.mediterraneense*	CBS 764.86	France	MH862045	MH873732	Vu et al. (2019)
*S.mediterraneense*	CBS 765.86	France	MH862046	MH873733	Vu et al. (2019)
*S.mediterraneense*	CBS 766.86	France	MH862047	MH873734	Vu et al. (2019)
*S.microspermum*	CBS 238.87	Guadeloupe	MH862070	−	Vu et al. (2019)
*S.ochroleucum*	CBS 767.86	France	MH862048	−	Vu et al. (2019)
*S.ochroleucum*	CBS 768.86	France	MH862049	MH873735	Vu et al. (2019)
*S.ochroleucum*	CBS 126049	USA	MH864062	MH875517	Vu et al. (2019)
*S.phaeosarcum*	CBS 728.81	Cote d’Ivoire	MH861481	MH873205	Vu et al. (2019)
*S.portentosum*	CBS 503.48	Canada	MH856447	MH873220	Vu et al. (2019)
*S.pseudopraestans*	CBS 737.91	−	MH862322	MH873994	Vu et al. (2019)
*S.pseudopraestans*	CBS 738.91	−	MH862323	MH873995	Vu et al. (2019)
*S.pseudopraestans*	CBS 739.91	−	MH862324	MH873996	Vu et al. (2019)
*S.pseudopraestans*	CBS 740.91	−	MH862325	MH873997	Vu et al. (2019)
*S.pseudopraestans*	CBS 741.91	−	MH862326	MH873998	Vu et al. (2019)
*S.pseudopraestans*	CBS 742.91	−	MH862327	−	Vu et al. (2019)
*S.quintasianum*	CBS 749.86	Cote d’Ivoire	MH862031	MH873719	Vu et al. (2019)
*S.quintasianum*	CBS 750.86	−	MH862032	MH873720	Vu et al. (2019)
*S.quintasianum*	CBS 751.86	−	MH862033	−	Vu et al. (2019)
*S.renisporum*	CBS 771.86	Indonesia	MH862051	MH873738	Vu et al. (2019)
*S.renisporum*	CBS 772.86	Indonesia	MH862052	MH873739	Vu et al. (2019)
*S.subduriusculum*	MEL:2382679	Australia	KP013042	−	Rosenthal et al. (2017)
*S.subduriusculum*	He 3590	China	MK625571	MK625499	Li et al. (2023)
*S.subduriusculum*	He 4146	Thailand	MK625570	MK625498	Li et al. (2023)
*S.yunnanense*	CLZhao 10758	China	MT611445	−	Wang et al. (2020)
*S.yunnanense*	CLZhao 10802	China	MT611446	−	Wang et al. (2020)
*S.yunnanense*	CLZhao 11010	China	MT611447	−	Wang et al. (2020)
*S.* sp. 1	UC2022985	USA	KP814265	−	Rosenthal et al. (2017)
*S.* sp. 1	UC2022946	USA	KP814564	−	Rosenthal et al. (2017)
*S.* sp. 2	MEL:2382745	Australia	KP012928	−	Rosenthal et al. (2017)
*S.* sp. 3	LR-40	Chile	MT366713	−	Direct Submission
*S.* sp. 4	Het 803-1	USA	OL989828	−	Otto et al. (2021)
*S.* sp. 4	NO 6-1-B	USA	OK173822	−	Otto et al. (2021)
*S.* sp. 4	iNAT:30809947	USA	MZ267776	−	Direct Submission
*S.* sp. 5	UoA SVB-F86	−	MT975590	−	Direct Submission
*S.* sp. 5	UC2023098	Canada	KP814402	−	Rosenthal et al. (2017)
